# miR-342-3p Inhibits Acute Myeloid Leukemia Progression by Targeting SOX12

**DOI:** 10.1155/2022/1275141

**Published:** 2022-09-08

**Authors:** Ying Wang, Xiaonan Guo, Lihua Wang, Lina Xing, Xiaolei Zhang, Jinhai Ren

**Affiliations:** Department of Hematology, The Second Hospital of Hebei Medical University, Shijiazhuang, 050000 Hebei, China

## Abstract

**Background:**

It is well known that microRNAs (miRNAs) interfere with the progression of various human malignancies. This article is aimed at exploring the regulating role of miR-342-3p in acute myeloid leukemia (AML) and its mechanism.

**Methods:**

We used the Gene Expression Omnibus (GEO) database to determine miR-342-3p differential expression patterns in AML patients' plasma and cells as well as healthy individuals' plasma and T cells. Quantitative real-time PCR and Western blotting were performed for plasma and cell miR-342-3p and SRY-related high-mobility-group box (SOX12) expression quantification, and cell counting kit-8 assay and flow cytometry were used for the determination of AML cell growth, cycle, and apoptosis. A dual-luciferase reporter gene assay was further carried out to identify the targeted association between miR-342-3p and SOX12 mRNA 3′UTR after prediction by a bioinformatics website. Pearson's correlation analysis was performed to analyze the connection between miR-342-3p and SOX12 expressions. The LinkedOmics database was utilized to explore the downstream pathways in which SOX12 was enriched.

**Results:**

Evidently downregulated plasma miR-342-3p and markedly elevated SOX12 were observed in AML patients versus healthy individuals. miR-342-3p mimics suppressed AML cell growth, enhanced apoptosis, and induced G0/G1 phase arrest; conversely, enhanced capacity of AML cells to proliferate, suppressed apoptosis, and accelerated cell cycle were observed after treatment with miR-342-3p inhibitors. SOX12 was confirmed as miR-342-3p's target gene. Overexpressing or knocking down SOX12 reversed miR-342-3p's impacts on AML cell growth, apoptosis, and cycle. Upregulated SOX12 was positively related to DNA replication and RNA polymerase signaling pathways.

**Conclusion:**

miR-342-3p affects apoptosis of AML cells and their ability to proliferate via targeted regulation of SOX12.

## 1. Introduction

Known as a highly heterogeneous and hematological malignancy, acute myeloid leukemia (AML) features undifferentiated bone marrow (BM) progenitor cells accumulated in the peripheral blood and BM [1]. Stem cell transplantation and chemotherapy remain the current mainstays of treatment for AML, but the treatment effect is unsatisfactory for those at advanced stages with recurrence and metastasis [2]. There are studies suggesting an association of gene mutations and abnormal expression of malignancy-related genes with the occurrence of AML [3, 4]. In this context, delving into possible mechanisms by which AML occurs and progresses is crucial for providing novel therapeutic targets for the disease.

As a kind of small endogenous noncoding RNAs widely present in eukaryotes that participate extensively in numerous biological processes (cell growth, differentiation, apoptosis, etc.) [5], microRNAs (miRNAs) are strongly linked to the genesis and development of multiple tumors, such as leukemia [6], gastric carcinoma [7], thyroid carcinoma [8], and hepatic carcinoma [9]. A large amount of research has indicated underexpressed miR-342-3p in lung carcinoma [10], pancreatic carcinoma [11], colon carcinoma [12], and other malignancies, with its expression highly correlated to disease staging, metastasis, and prognosis. For instance, underexpressed miR-342-3p in non-small-cell lung carcinoma is linked to poor patient outcomes, and overexpressing miR-342-3p markedly represses tumor cells' capacity to proliferate and migrate, demonstrating miR-342-3p's role as a candidate diagnostic biomarker and therapeutic target [13]. We learned through bioinformatics analysis that in AML patients, plasma miR-342-3p is abnormally underexpressed, yet it is still unclear about the mechanism underlying disease occurrence and development.

Being a member of the SOX gene family, SRY-related high-mobility-group box (SOX12) is a key transcription factor that is capable of maintaining the self-renewing and proliferation capacities of many tissue-specific stem cells (SCs) like embryonic SCs [14]. Over the past few years, increasing research has demonstrated the crucial role of SOX12 in tumor occurrence, growth, proliferation, invasion, and metastasis, with different biological behaviors in different types of tumors [15]. Reportedly, SOX12 is a tumor-promoting gene in hepatocellular cancer [16] and breast carcinoma [17]. However, in colorectal cancer [18], it is a tumor-suppressing gene. There has also been research suggesting that SOX12 is significantly highly expressed in AML, and SOX12 knockdown represses THP1 cell viability, reduces cell colonies, and induces G1 phase cell cycle arrest, indicating that SOX12 can be a new potential target for AML [15]. Bioinformatics prediction showed that SOX12 may be miR-342-3p's target. It is found that miR-342-3p overexpression in liver cancer SCs leads to reduced tumor volume, as well as lower Sox2 at mRNA and protein levels [19]. Due to the unknown mechanism of miR-342-3p and its target SOX12 in AML, the motivation and novelty of this research is to survey the possible mechanisms by which miR-342-3p influences AML cell multiplication and apoptosis via targeting SOX12, aiming to decipher new mechanisms of AML genesis and development and provide a theoretical basis for seeking novel targeted therapies.

## 2. Data and Methods

### 2.1. Collection of Plasma Samples

Forty-seven AML plasma samples collected in this experiment were obtained from patients hospitalized between February 2018 and May 2020. All AML cases were confirmed by BM cell morphology and FCM examinations. The control group consisted of 47 healthy pregnant women who had normal full-term delivery in the hospital during the same period, from whom umbilical cord blood samples were collected. The current research was endorsed by the Hospital's Ethics Committee and conducted following the *Declaration of Helsinki* and the ethics committee guidelines. Every participant signed informed consent.

### 2.2. Cultivation and Transfection of Cells

The AML cells (HL-60, THP-1, KG-1, CCRF-CEM, and MOLT-3) used in this study were all supplied by the American Type Culture Collection (Manassas, USA). First, we selected healthy people undergoing physical examinations in the hospital. Next, Ficoll liquid (Solarbio, Beijing, China) was used to separate their BM primary leukocytes. The culture medium of KG-1 and HL-60 was 10% fetal bovine serum supplemented Roswell Park Memorial Institute Medium 1640 (RPMI1640; both from Gibco, Carlsbad, CA, USA), which was placed in a 5% CO_2_, 37°C incubator. When the cells grew to about 90% confluency, they were subjected to 0.25% trypsin (Roche, Basel, Switzerland) digestion, centrifugation, and passage. This was followed by trypsinization of logarithmic-phase KG-1 and HL-60 cells and their subsequent inoculation at 1 × 10^6^ cells/well into the wells of 6-well plates. After being incubated overnight, they were transfected with the following oligonucleotides and plasmids (all from RiboBio, Guangzhou, China): miR-342-3p mimics (miR mimics), mimics control (mimics NC), inhibitors (miR inhibitors), and negative control inhibitors NC, as well as SOX12 and si-SOX12, following the instructions of the Lipofectamine™ 2000 (Invitrogen, Carlsbad, CA, USA) transfection reagent. Quantitative real-time polymerase chain reaction (qRT-PCR) determined the transfection efficiency 48 h after transfection for further analysis.

### 2.3. qRT-PCR

TRIzol (Invitrogen, Shanghai, China) extracted cell and tissue total RNA, after which reverse transcription of the total RNA into cDNA was carried out employing a reverse transcription kit ordered from Qiagen, Hilden, Germany. qRT-PCR was then carried out with the use of a SYBR® Premix Ex Taq™ (Takara, Kusatsu, Shiga, Japan) and the ABI7500 system (Applied Biosystems, San Francisco, CA, USA). SOX12 mRNA and miR-342-3p levels, relative to U6 and GAPDH, respectively, were quantified by the 2^-*ΔΔ*Ct^ formula. Three independent replicates were performed for each experiment. The following are the primer sequences: miR-342-3p sense (5′-3′): GGGTCTCACACAGAAATCGC and antisense (5′-3′), CAGTGCGTGTCGTGGAGT; SOX12 sense: AGGAAGGTGAAGAGGAGACG and antisense, ATCATCTCGGTAACCTCGGG; GAPDH sense, CACCCACTCCTCCACCTTTG and antisense, CCACCACCCTGTTGCTGTAG; and U6 sense: CGCGCTTCGGCAGCACATATACT and antisense, ACGCTTCACGAATTTGCGTGTC.

### 2.4. Cell Counting Kit-8 (CCK-8) Assay

KG-1 and HL-60 transfected 48 h later were inoculated at 2000 cells/well in 96-well plates. We set 5 replicate wells, into which 10 *μ*L/well CCK-8 solution (Invitrogen, Shanghai, China) was placed on days 1, 2, and 3 for another 2 h of cultivation in the incubator. Each well's absorbance (OD)_450 nm_ was determined using a microplate reader (Multiskan, Thermo, USA).

### 2.5. Flow Cytometry (FCM)

We followed the manufacturer's instructions of Annexin V Apoptosis Detection Kit I and PI (BD Biosciences) to measure cell apoptosis. For each group of transfected cells, they were suspended in a 500 *μ*L binding buffer and then mixed with Annexin V-FITC/PI double-labeled staining reagent (5 *μ*L). In the dark, the mixture was treated with ice incubation and PI solution (5 *μ*L) cultivation with, both for 20 min. Cell detection was performed within 1 h with a flow cytometer. A Cell Cycle and Apoptosis Analysis Kit was utilized to determine the cell cycle. In brief, the collected cells were treated with a PI-RNase mixed solution, and FCM (BD FACSCanto™ II) was carried out to for fluorescence analysis. For each sample, at least 10^4^ cells were required for analysis. Each experiment was repeated 3 times.

### 2.6. Dual-Luciferase Reporter (DLR) Gene Assay

A Lipofectamine™ 2000 kit was utilized to cotransfect wild-type (WT) or mutant (MUT) DLR vectors (WT-SOX12 or MUT-SOX12; RiboBio, Guangzhou, China) and miR mimics or mimics NC into KG-1 cells and to cotransfect WT-SOX12 or MUT-SOX12 and miR inhibitors or inhibitors NC into HL-60. Luciferase activity was determined 48 h later under the instructions of the DLR gene detection kit (Promega, Madison, WI, USA). The ratio of the luminescence intensity of ranilla luciferase/firefly luciferase was the binding intensity of SOX12 to miR-342-3p.

### 2.7. Western Blot (WB) Analysis

Cell culturing was terminated 48-72 h posttransfection, after which total protein isolation from cells was conducted using RIPA lysis buffer (Pierce, Rockford, IL, USA) +1% protease inhibitors. Samples with an equal amount were isolated via polyacrylamide gel electrophoresis (10%) and then electrotransferred onto a PVDF membrane. Following 1 h of 5% skim milk blocking on a shaker at an ambient temperature, it was incubated with an anti-SOX12 primary antibody (Product No.: SAB1412152; dilution: 1 : 1000; Sigma-Aldrich; Merck KGaA) overnight at 4°C on a shaker. After three rinses in TBST, it was incubated (1 h) with a secondary antibody anti-GAPDH antibody (Product No.: ab6789; Abcam, Shanghai, China) diluted at 1 : 5000, also at an ambient temperature. After 3 rinses in TBST and development, the protein bands were analyzed by a gel imaging system. The target protein's relative expression = OD_target protein_/OD_internal control_.

### 2.8. Pathway Enrichment Analysis

Further, we performed KEGG pathway enrichment analysis using DAVID to explore the biological functional roles of the downstream target gene set of miR-342-3p.

### 2.9. Gene Set Enrichment Analysis (GSEA)

Samples were grouped (high/low expression groups) based on median SOX12 expression value, and the influences of gene expression profiling on tumors were investigated by GSEA (screening criteria: FDR < 0.25 and *P* < 0.05).

### 2.10. Statistics and Analysis

The statistical processing and drawing software were SPSS 22.0 (SPSS Inc., Chicago, IL, USA) and GraphPad Prism 9 software (GraphPad Software, La Jolla, CA), respectively. The limma package analyzed the differential expression of each dataset. |log(*FC*)| ≥1 and adj. *P* value < 0.05 were used as the screening criteria for differentially expressed genes (DEGs). The results of all measurement experiments, denoted by mean ± standard deviation, were compared between groups and among multiple groups via independent sample *t*-test and single factor analysis of variance, respectively, with *P* < 0.05 indicating a difference of statistical significance.

## 3. Results

### 3.1. Plasma and Cell miR-342-3p in AML Patients

**Figure 1:**
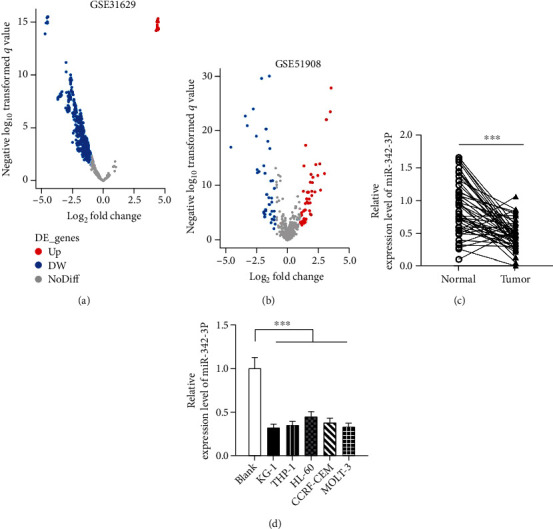
Plasma and cell miR-342-3p expression in AML patients. (a and b) Microarray datasets, downloaded from the GEO database, were utilized to analyze miRNAs' expression differences, and the volcano plots display differentially expressed miRNAs in AML plasma samples. (c) miR-342-3p expression in 47 cases of AML plasma and normal plasma by qRT-PCR. (d) miR-342-3p expression in primary leukocytes and AML cell lines (HL-60, THP-1, KG-1, CCRF-CEM, and MOLT-3) by qRT-PCR. ^∗∗∗^*P* < 0.001.

To understand miR-342-3p expression characteristics, we used the Gene Expression Omnibus (GEO) database (URL: https://www.ncbi.nlm.nih.gov/gds/) to download gene chip datasets GSE31629 [20] and GSE51908 [21, 22] to analyze changes in plasma and cell miRNA expression in AML patients. In the GSE31629 dataset, 771 downregulated and 17 upregulated miRNAs were identified; in the dataset GSE51908, 40 miRNAs were downregulated and 47 were upregulated (Figures 1(a) and 1(b)). Subsequently, we utilized qRT-PCR to quantify miR-342-3p levels in AML patients' plasma and found remarkably downregulated expression in AML patients as compared to the normal group (Figure 1(c)). Similarly, miR-342-3p expression in AML cell strains (THP-1, HL-60, KG-1, CCRF-CEM, and MOLT-3) was dramatically reduced compared with primary leukocytes (Figure 1(d)).

### 3.2. Biological Functions of miR-342-3p in AML

**Figure 2:**
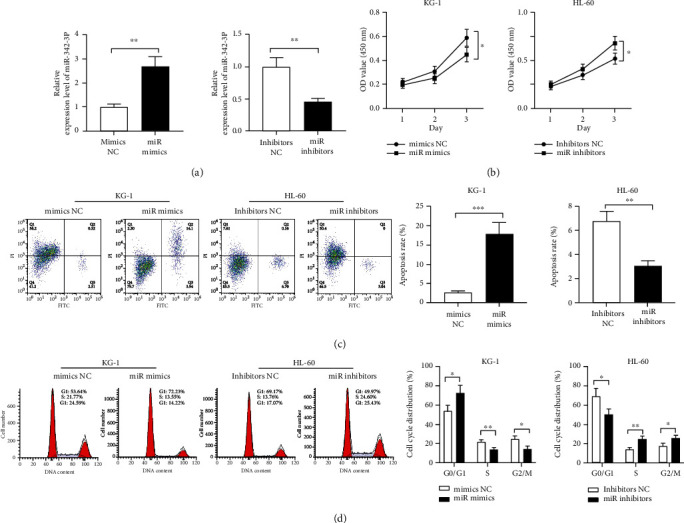
Impacts of miR-342-3p on AML cell growth, apoptosis, and cycle. (a) qRT-PCR was performed to verify transfection efficiency after transfecting mimics NC and miR mimics into KG-1 and inhibitors NC and miR inhibitors into HL-60. (b) CCK-8 assay was carried out posttransfection to determine KG-1 and HL-60 cell viability. (c and d) Flow cytometry was conducted to measure posttransfection KG-1 and HL-60 cell apoptosis and cycle changes. ^∗^*P* < 0.05, ^∗∗^*P* < 0.01, and ^∗∗∗^*P* < 0.001.

miR-342-3p showed the lowest expression in KG-1 and the highest expression in HL-60 among the five AML cell strains. Therefore, we transfected KG-1 with miR-342-3p mimics and mimics NC and HL-60 with miR-342-3p inhibitors and inhibitors NC, separately. qRT-PCR verified the success of transfection (Figure 2(a)). The changes in cell viability were examined through CCK-8 assay. It showed dramatically suppressed KG-1 cell viability by miR-342-3p mimics and markedly enhanced HL-60 viability by miR-342-3p inhibitors, versus the control group (Figure 2(b)). Next, we performed FCM to determine miR-342-3p's impacts on cell apoptosis and cycle. As demonstrated by Figures 2(c) and 2(d), enhanced apoptosis and blocked cell cycle were observed following miR-342-3p mimic intervention, while restrained cell apoptosis and accelerated cell cycle were found after miR-342-3p inhibitors transfection.

### 3.3. SOX12 Is miR-342-3p's Direct Downstream Target

**Figure 3:**
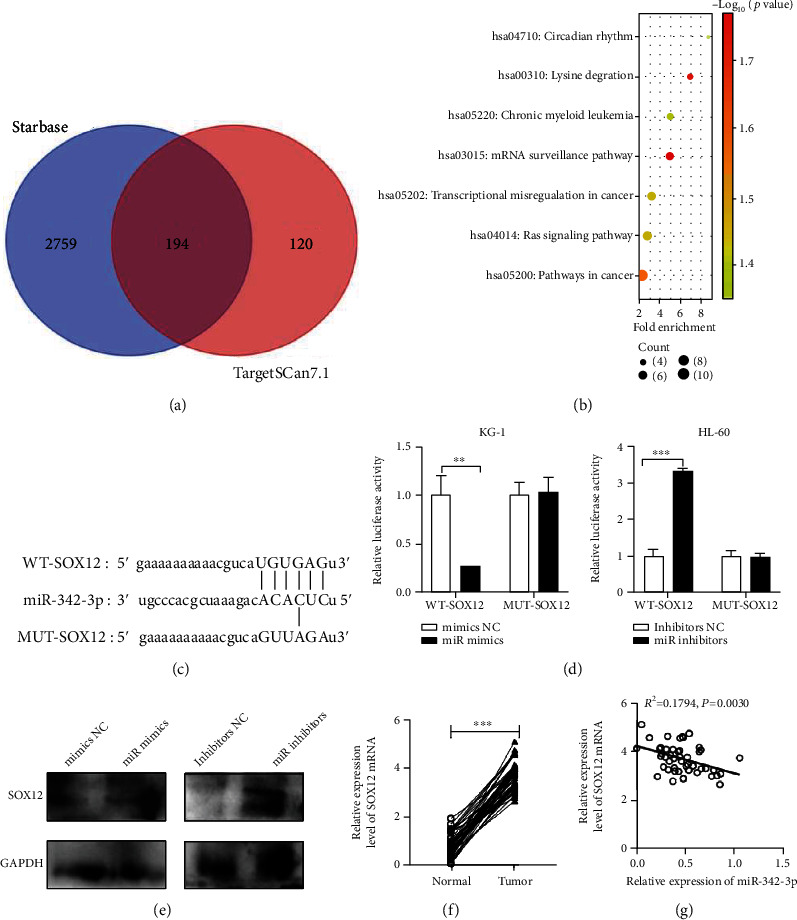
SOX12 is miR-342-3p's direct downstream target. (a) StarBase (URL: http://starbase.sysu.edu.cn/index.php) and TargetScan (URL: TargetScanHuman 7.2) were employed to predict miR-342-3p's downstream targets and a Venn diagram was drawn. (b) Pathway enrichment analysis of miR-342-3p's downstream target gene sets using the KEGG database. (c) The binding sequences of SOX12 mRNA 3′UTR WT and MUT to miR-342-3p. (d) Impacts of miR-342-3p overexpression on WT-SOX12 and MUT-SOX12 luciferase activity by dual-luciferase reporter gene assay. (e) Impacts of miR-342-3p mimics or inhibitors on SOX12 protein expression by Western blot. (f) qRT-PCR detection of SOX12 mRNA expression in the plasma of 47 AML patients and healthy individuals. (g) Pearson correlation analysis of the correlation between SOX12 mRNA and miR-342-3p expression in AML plasma. ^∗^*P* < 0.05, ^∗∗^*P* < 0.01, and ^∗∗∗^*P* < 0.001.

To delve deeper into the mechanism by which miR-342-3p affects AML, we performed downstream target prediction of miR-342-3p utilizing bioinformatics websites TargetScan (TargetScanHuman 7.2) and StarBase (URL: http://starbase.sysu.edu.cn/index.php) (Figure 3(a)), as well as enrichment analysis of miR-342-3p's downstream target genes using the Kyoto Encyclopedia of Genes and Genomes (KEGG) database. The results identified strong correlations of the above target genes with the Ras signaling pathway and the mRNA surveillance pathway (Figure 3(b)). SOX12 is an important target gene, and there are binding loci between miR-342-3p and SOX12 mRNA 3′UTR, which is shown in Figure 3(c). Upregulating miR-342-3p remarkably suppressed WT-SOX12's luciferase activity, and inhibiting miR-342-3p statistically enhanced WT-SOX12's luciferase activity, versus the control group, as indicated by the DLR gene assay; there was no significant effect on that of the MUT-SOX12 group (Figure 3(d)). WB indicated that miR-342-3p mimics can lower SOX12 expression, and conversely, miR-342-3p inhibitors can elevate SOX12 expression (Figure 3(e)). Through qRT-PCR, we found noticeably increased SOX12 mRNA expression in the AML group versus the normal group (Figure 3(f)) and an inverse connection between miR-342-3p expression and SOX12 mRNA (Figure 3(g)).

### 3.4. SOX12 Can Reverse miR-342-3p's Impacts on AML

**Figure 4:**
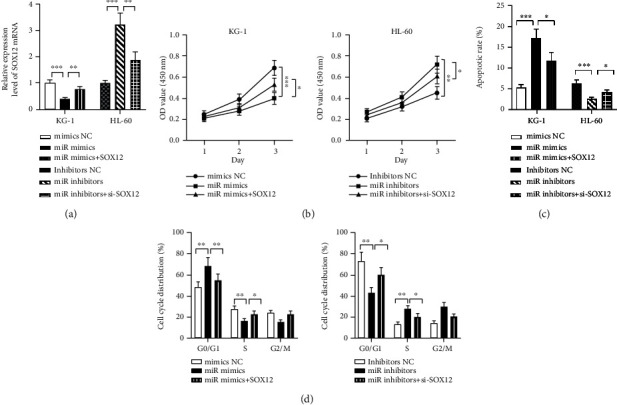
SOX12 can reverse miR-342-3p's impacts on AML cell growth, apoptosis, and cycle. (a) qRT-PCR was performed to verify transfection efficiency after transfecting miR mimics+SOX12 into KG-1 and miR inhibitors and si-SOX12 into HL-60. (b) KG-1 and HL-60 cell viability detected by CCK-8 assay posttransfection. (c and d) KG-1 and HL-60 cell apoptosis and cycle changes measured by flow cytometry posttransfection. ^∗^*P* < 0.05, ^∗∗^*P* < 0.01, and ^∗∗∗^*P* < 0.001.

Further, to probe into the influences of SOX12 and miR-342-3p on AML cell growth, apoptosis, and cycle, we cotransfected miR mimics, mimics NC, SOX12 and miR inhibitors, inhibitor NC, and si-SOX12 into KG-1 and HL-60 cells, respectively. qRT-PCR validated the success of all transfections (Figure 4(a)). Then, according to CCK-8 assay and FCM, miR-342-3p mimics dramatically suppressed the ability of cells to proliferate, induced cell apoptosis, and blocked cell cycle processes versus control cells, whereas SOX12 overexpression reversed these effects (Figures 4(b)–4(d)). miR-342-3p inhibitors markedly facilitated cell growth, inhibited apoptosis, and accelerated cell cycle processes, while knocking down SOX12 restored these effects (Figures 4(b)–4(d)).

### 3.5. SOX12 Activates DNA Replication and RNA Polymerase Signaling Pathways

**Figure 5:**
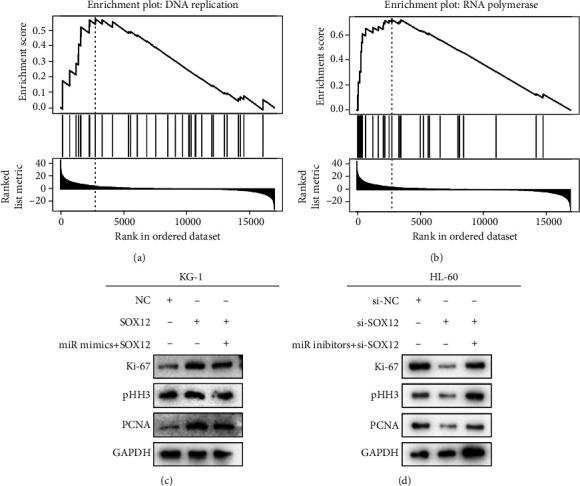
SOX12 activates DNA replication and RNA polymerase signaling pathways. (a and b) The GSEA plot shows a positive connection between SOX12 expression and DNA replication and RNA polymerase pathways. (c and d) Western blot analysis of Ki-67, pHH3, and PCNA expressions.

GSEA was further employed for signaling pathway enrichment analysis, so as to elucidate the mechanism underlying SOX12's regulation of AML cells' biological functions; it identified that high SOX12 expression was positively related to DNA replication and the activation of RNA polymerase signaling pathways (Figures 5(a) and 5(b)). Next, WB was employed to detect the roles played by SOX12 and miR-342-3p in DNA replication and the expression of cell cycle-related proteins (Ki-67, pHH3, and PCNA) (Figures 5(c) and 5(d)). It was suggested that SOX12 overexpression led to enhanced expression of pHH3, Ki-67, and PCNA, and SOX12 knockdown resulted in decreased expression of these genes, while miR-342-3p mimics and inhibitors reversed the above mentioned effects, respectively. All the evidence demonstrates the inhibitory action of miR-342-3p/SOX12 against DNA replication and cell cycle signaling axis.

## 4. Discussion

AML, primarily manifesting as anemia, bleeding, fever, and infection, is a hematopoietic malignancy with high cytogenetic or molecular heterogeneity that is clinically characterized by acute and severe onset, difficulty in control, easy relapse, and poor prognosis, which seriously threaten human life and health [23, 24]. According to cell morphological and histochemical characteristics, AML can be classified as FAB, MIC, and other types [23, 24]. Except for acute promyelocytic leukemia, the complete response rate of combined chemotherapy for AMI is only 65-75%, and the long-term disease-free survival rate (leukemia-free survival) is 20-30%; the five-year survival is only 40% for those aged under 60 [25, 26]. Despite dramatic advances in the treatment of AML, there are still quite a few AML patients who have a poor prognosis. To improve patients' outcomes, it is highly significant to delve into the nosogenesis of AML and its treatment.

miRNAs, as a kind of highly conserved 20-24 nucleotide length-long noncoding RNAs, are capable of binding to the incomplete complementary locus in mRNA 3′UTR to inhibit gene expression after transcription [27]. As cancer promoters or tumor suppressors, they can participate in modulating multiple biological processes (cell cycle progression, growth, apoptosis, etc.) in malignancies [28]. Evidence in recent years has revealed that miRNAs are strongly linked to AML occurrence and development and play a crucial regulatory role in AML occurrence by regulating their target genes. There is a study indicating that in AML, miR-12462 is low-expressed and inhibits AML cell growth both in vivo and in vitro and enhances AML cell chemotherapy sensitivity via targeting SLC9A1, thus playing a tumor-suppressing role [29]. miR-212-5p keeps at a low level in AML cells, and overexpressing miR-212-5p can suppress AML cell growth and promote apoptosis [30]. miR-342-3p, once called miR-342, is a 23-base single-stranded small RNA that is located on chromosome 14q32. miR-342-3p is located in the intron of the EVL gene, its host gene. A number of experiments have shown that miR-342-3p shows consistent expression with EVL's mRNA expression [31]. The involvement of miR-342-3p in cancer occurrence and development as a tumor suppressor and kinase regulator has been well documented. For example, miR-342-3p, expressing at low levels in hepatocellular carcinoma, restrains the capacities of tumor cells to growth and migrate through negative regulation of MCT1 [32]. In glioma tissues and cells, miR-342-3p is regulated by circ_ARF1, forming a feedback loop composed of U2AF2, ARF1, ISL2, and miR-342-3p, which modulates glioma development [33]. In ovarian cancer tissues and cell lines, underexpressed miR-342-3 is linked to patients' poor clinicopathological markers; miR-342-3 overexpression inhibits tumor cell migration and invasion [34]. miR-342-3p underexpression in acute leukemia [35] and a strong correlation of miR-342-3p expression with the therapeutic effect of acute leukemia and chronic leukemia have also been demonstrated [36, 37]. This study first discovered downregulated miR-342-3p in AML patient's plasma. Further research suggested that miR-342-3p mimics suppressed AML cell growth, accelerated apoptosis, and induced G0/G1 cell cycle arrest, while inhibiting miR-342-3p contributed to the opposite effects.

SOX12 is a member of the SOX family, which is a newly discovered supergene family to encode transcription factors, and is mainly characterized by its product having an HMG-box conserved motif that can specifically bind to DNA sequences to modulate other genes' expression, thereby playing an important part in animal development and the maintenance of cell characteristics [38, 39]. Recent evidence has also revealed that the SOX gene family members may feature prominently in cell proliferation and even cancerization and the stage transformation of malignancies; moreover, the mutation, deletion, and overexpression of SOX genes are strongly linked to the genesis and development of various malignancies [40]. There are studies demonstrating the correlation of SOX12 expression dysregulation with the occurrence and development of colon carcinoma [41], squamous cell carcinoma of the esophagus [42], renal carcinoma [43], and AML [44]. Research has indicated that after SOX12 knockdown, *β*-catenin mRNA, and protein levels are dramatically reduced, and TCF/Wnt activity is reduced, which leads to G1 phase cell cycle arrest and decreased cell colonies, indicating that SOX12 may regulate *β*-catenin expression to interfere with the TCF/Wnt pathway and participate in leukemia progression [15]. SOX12 is significantly highly expressed in AML cells, and miR-625-5p targets SOX12 directly to suppress AML cell growth and apoptosis [45]. Additionally, SOX12 knockdown inhibits AML cell proliferation via repressing Wnt/*β*-catenin axis activity, whereas miR-489-3p can reverse the effect of SOX12 [44], which is similar to our observations. We discovered the role of SOX12 as a downstream target of miR-342-3p, and that miR-342-3p inactivates the DNA replication pathway through targeted regulation of SOX12, thus suppressing the growth of AML cells while inducing apoptosis and G0/G1 cell cycle arrest. However, there is still room for improvement in this study. Although we found that miR-342-3p inactivates the DNA replication pathway through targeted regulation of SOX12, some other downstream pathways were not further investigated. Furthermore, besides SOX12, there may be other important downstream targets of miR-342-3p that we have missed. Thus, more experiments are needed to further reveal the mechanisms underlying the action of miR-342-3p against AML.

## 5. Conclusion

To sum up, miR-342-3p mimics can suppress AML cell proliferation. Furthermore, miR-342-3p negatively regulates SOX12 expression at the protein and mRNA levels, thereby modulating DNA replication signaling pathways and ultimately inhibiting cell proliferation. All these suggest that miR-342-3p and SOX12 may be targets for AML treatment.

## Data Availability

The labeled datasets used to support the findings of this study are available from the corresponding author upon request.

## References

[1] De Kouchkovsky I., Abdul-Hay M. (2016). Acute myeloid leukemia: a comprehensive review and 2016 update. *Blood Cancer Journal*.

[2] Infante M. S., Piris M. Á., Hernández-Rivas J. Á. (2018). Alteraciones moleculares en leucemia mieloide aguda y sus implicaciones clinicas y terapeuticas. *Medicina Clínica (English Edition)*.

[3] Elgarten C. W., Aplenc R. (2020). Pediatric acute myeloid leukemia: updates on biology, risk stratification, and therapy. *Current Opinion in Pediatrics*.

[4] Prada-Arismendy J., Arroyave J. C., Röthlisberger S. (2017). Molecular biomarkers in acute myeloid leukemia. *Blood Reviews*.

[5] Zhang L., Liao Y., Tang L. (2019). Microrna-34 family: a potential tumor suppressor and therapeutic candidate in cancer. *Journal of Experimental & Clinical Cancer Research*.

[6] Liu L., Ren W., Chen K. (2017). Mir-34a promotes apoptosis and inhibits autophagy by targeting hmgb1 in acute myeloid leukemia cells. *Cellular Physiology and Biochemistry*.

[7] Hu S., Zheng Q., Wu H., Wang C., Liu T., Zhou W. (2017). Mir-532 promoted gastric cancer migration and invasion by targeting nkd1. *Life Sciences*.

[8] Bian S. (2020). miR-4319 inhibited the development of thyroid cancer by modulating FUS- stabilized SMURF1. *Journal of Cellular Biochemistry*.

[9] Fang T., Lv H., Lv G. (2018). Tumor-derived exosomal mir-1247-3p induces cancer-associated fibroblast activation to foster lung metastasis of liver cancer. *Nature Communications*.

[10] Shen Q., Sun Y., Xu S. (2020). Linc01503/mir-342-3p facilitates malignancy in non-small-cell lung cancer cells via regulating lasp1. *Respiratory Research*.

[11] Meng X., Ma J., Wang B., Wu X., Liu Z. (2020). Long non-coding RNA OIP5-AS1 promotes pancreatic cancer cell growth through sponging mir-342-3p via AKT/ERK signaling pathway. *Journal of Physiology and Biochemistry*.

[12] Zhou L., Li J., Tang Y., Yang M. (2021). Exosomal LncRNA LINC00659 transferred from cancer-associated fibroblasts promotes colorectal cancer cell progression via miR-342-3p/ANXA2 axis. *Journal of Translational Medicine*.

[13] Xue X., Fei X., Hou W., Zhang Y., Liu L., Hu R. (2018). Mir-342-3p suppresses cell proliferation and migration by targeting AGR2 in non-small cell lung cancer. *Cancer Letters*.

[14] Zou S., Wang C., Liu J. (2017). Sox12 is a cancer stem-like cell marker in hepatocellular carcinoma. *Molecules and Cells*.

[15] Wan H., Cai J., Chen F., Zhu J., Zhong J., Zhong H. (2017). SOX12: a novel potential target for acute myeloid leukaemia. *British Journal of Haematology*.

[16] Yuan P., Meng L., Wang N. (2017). SOX12 upregulation is associated with metastasis of hepatocellular carcinoma and increases CDK4 and IGF2BP1 expression. *European Review for Medical and Pharmacological Sciences*.

[17] Xu J., Zhang J., Li L., Mao J., You T., Li Y. (2020). SOX12 expression is associated with progression and poor prognosis in human breast cancer. *American Journal of Translational Research*.

[18] Li J., Zheng Y., Li X. (2020). UCHL3 promotes proliferation of colorectal cancer cells by regulating SOX12 via AKT/mTOR signaling pathway. *American Journal of Translational Research*.

[19] Xu C., Sun W., Liu J., Pu H., Li Y. (2022). MiR-342-3p inhibits LCSC oncogenicity and cell stemness through HDAC7/PTEN axis. *Inflammation Research*.

[20] Yamagishi M., Nakano K., Miyake A. (2012). Polycomb-mediated loss of miR-31 activates NIK-dependent NF-*κ*B pathway in adult T cell leukemia and other cancers. *Cancer Cell*.

[21] Tan Y. S., Kim M., Kingsbury T. J., Civin C. I., Cheng W.-C. (2014). Regulation of RAB5C is important for the growth inhibitory effects of MiR-509 in human precursor-B acute lymphoblastic leukemia. *PLoS One*.

[22] Candia J., Cherukuri S., Guo Y. (2015). Uncovering low-dimensional, miR-based signatures of acute myeloid and lymphoblastic leukemias with a machine-learning-driven network approach. *Convergent Science Physical Oncology*.

[23] Bose P., Vachhani P., Cortes J. E. (2017). Treatment of relapsed/refractory acute myeloid leukemia. *Current Treatment Options in Oncology*.

[24] Estey E. H. (2018). Acute myeloid leukemia: 2019 update on risk-stratification and management. *American Journal of Hematology*.

[25] Döhner H., Weisdorf D. J., Bloomfield C. D. (2015). Acute myeloid leukemia. *New England Journal of Medicine*.

[26] DiNardo C., Lachowiez C. (2019). Acute myeloid leukemia: from mutation profiling to treatment decisions. *Current Hematologic Malignancy Reports*.

[27] Simonson B., Das S. (2015). MicroRNA therapeutics: the next magic bullet?. *Mini Reviews in Medicinal Chemistry*.

[28] Krol J., Loedige I., Filipowicz W. (2010). The widespread regulation of microRNA biogenesis, function and decay. *Nature Reviews Genetics*.

[29] Jia Y., Liu W., Zhan H.-E. (2020). Roles of hsa-miR-12462 and SLC9A1 in acute myeloid leukemia. *Journal of Hematology & Oncology*.

[30] Lin J., Zeng H., Zhao J. (2018). Mir-212-5p regulates the proliferation and apoptosis of AML cells through targeting FZD5. *European Review for Medical and Pharmacological Sciences*.

[31] Radfar M. H., Wong W., Morris Q. (2011). Computational prediction of intronic microRNA targets using host gene expression reveals novel regulatory mechanisms. *PLoS One*.

[32] Komoll R.-M., Hu Q., Olarewaju O. (2021). Microrna-342-3p is a potent tumour suppressor in hepatocellular carcinoma. *Journal of Hepatology*.

[33] Jiang Y., Zhou J., Zhao J. (2020). The U2AF2/circRNA ARF1/miR-342–3p/ISL2 feedback loop regulates angiogenesis in glioma stem cells. *Journal of Experimental & Clinical Cancer Research*.

[34] Wang C., Zhang W., Xing S., Wang Z., Wang J., Qu J. (2019). Mir-342-3p inhibits cell migration and invasion through suppressing forkhead box protein Q1 in ovarian carcinoma. *Anti-Cancer Drugs*.

[35] Coskun E., Neumann M., Schlee C. (2013). Microrna profiling reveals aberrant microrna expression in adult ETP-ALL and functional studies implicate a role for miR-222 in acute leukemia. *Leukemia Research*.

[36] Mosakhani N., Räty R., Tyybäkinoja A., Karjalainen-Lindsberg M.-L., Elonen E., Knuutila S. (2013). MicroRNA profiling in chemoresistant and chemosensitive acute myeloid leukemia. *Cytogenetic and Genome Research*.

[37] Li S., Moffett H. F., Lu J. (2011). MicroRNA expression profiling identifies activated B cell status in chronic lymphocytic leukemia cells. *PLoS One*.

[38] Grimm D., Bauer J., Wise P. (2020). The role of sox family members in solid tumours and metastasis. *Presented at Seminars in Cancer Biology*.

[39] Sarkar A., Hochedlinger K. (2013). The sox family of transcription factors: versatile regulators of stem and progenitor cell fate. *Cell Stem Cell*.

[40] Ashrafizadeh M., Taeb S., Hushmandi K. (2020). Cancer and sox proteins: new insight into their role in ovarian cancer progression/inhibition. *Pharmacological Research*.

[41] Du F., Chen J., Liu H. (2019). SOX12 promotes colorectal cancer cell proliferation and metastasis by regulating asparagine synthesis. *Cell Death & Disease*.

[42] Li C., Zhu M., Zhu J. (2021). SOX12 contributes to the activation of the JAK2/STAT3 pathway and malignant transformation of esophageal squamous cell carcinoma. *Oncology Reports*.

[43] Chen Z., Xiao K., Chen S., Huang Z., Ye Y., Chen T. (2020). Circular RNA hsa_circ_001895 serves as a sponge of microRNA-296-5p to promote clear cell renal cell carcinoma progression by regulating SOX12. *Cancer Science*.

[44] Li C., Gao Q., Wang M., Xin H. (2021). LncRNA SNHG1 contributes to the regulation of acute myeloid leukemia cell growth by modulating miR-489-3p/SOX12/Wnt/*β*-catenin signaling. *Journal of Cellular Physiology*.

[45] Shang Z., Ming X., Wu J., Xiao Y. (2021). Downregulation of circ 0012152 inhibits proliferation and induces apoptosis in acute myeloid leukemia cells through the miR-625-5p/sox12 axis. *Hematological Oncology*.

